# Fellow-Workers and Their Doings

**Published:** 1914-01-17

**Authors:** 


### ROUND THE WORLD.

### Fellow-Workers and their Doings.

[The Editor Invites Early Information and Contributions Marked "Fellow-Workers."]

Dr. Frederick Lucien Golla, M.D., M.R.C.P., has been appointed assistant physician to the Hospital for Epilepsy and Paralysis in Maida Vale. Dr. Golla, who is one of the out-patient physicians at St. George's, has for some years given much of his time to neurology, being formerly house physician and resident medical officer at the "National Hospital" in Queen Square, whence he went to the West-End Hospital for Nervous Diseases, in Welbeck Street, where he holds the post of physician.

Dr. Edwin Greaves Fearnsides, M.B., M.R.C.P., one of the medical registrars at the London Hospital, has been also appointed assistant physician to the Maida Vale Hospital for Paralysis and Epilepsy, on the staff of which he will be immediately junior to Dr. Golla.

Guy's has lately provided the Leeds Public Dispensary with a new ophthalmic surgeon in the person of Mr. Harry Lee, M.B., F.R.C.S., who has resigned his post of chief ophthalmic clinical assistant at the first-named institution. Mr. Lee was at Cambridge before he went to Guy's, and has worked also as clinical assistant in the Ophthalmological Department of the West London Hospital.

Dr. Arthur Arbuthnot Straton, who has recently been successful in obtaining a University gold medal at the M.D. Examination of London University, at which he gained distinctions in Branch IV. (midwifery and diseases of women), is a former student of St. Mary's Hospital, where he has lately been resident obstetric officer. Dr. Straton is also an F.R.C.S. Edin., and has served as resident surgical officer to the Wolverhampton General Hospital, and as senior house surgeon to the Sussex County Hospital at Brighton.

In spite of much time devoted to recent work with radium, Dr. Robert Knox is continuing his very instructive investigations at the Cancer Hospital in regard to lung metastases. Prognosis in cancer often depends so much on the question as to whether the lungs have or have not become invaded, that reliable *x*-ray methods of settling it are invaluable to both surgeon and general practitioner. The practical usefulness of the results so far obtained by Dr. Knox is clearly shown by the excellent series of mounted slides at the hospital, which by an ingenious system of illumination can be demonstrated to visitors at a moment's notice. The successful detection of early lung deposits which has been secured in this way depends largely on the perfection of a method of rapid *x*-ray photography, and calls for the services of specially trained assistants.

Mr. L. J. Austin, M.C., F.R.C.S., a London Hospital man, has been appointed surgeon to the Cambridge Hospital for the study of special diseases. From small beginnings this institution, which is given up to the particular investigation of pathological problems and retains its inmates for as long as may be necessary for the investigation of their maladies, has achieved a position of considerable importance in the world of scientific research. It is now considered that the efficiency of this research hospital will be further secured by the development of a surgical department, to which end an operating theatre is being built. Mr. Austin's appointment is interesting as being the first of its kind made at this institution. The new member of its staff was formerly surgical registrar at the "London," and has lately been directing one of the special Ear Clinics under the London County Council.

By securing the appointment of physician to out-patients at St. Mary's Hospital, Dr. Frederick Langmead, M.D., F.R.C.P., who has for the past year or two been a member of the staff at the Royal Free Hospital, returns to his Alma Mater, where a few years ago he did yeoman service as registrar and casualty physician. Dr. Langmead, who is to be congratulated on his early attainment of the coveted F.R.C.P., has devoted his scientific investigations chiefly to diseases of children, and carries on the tradition which St. Mary's has had for many years past in connection with this branch, particularly in relation to problems of acute rheumatism. His work in this connection has been associated with two well-known special institutions—namely, the Great Ormond Street Hospital for Sick Children, where he holds the post of assistant physician, and the Paddington Green Children's Hospital, where he was at one time physician to out-patients.

The common errors of neurological diagnosis collected by Dr. S. A. Kinnier Wilson, of the National Hospital for the Paralysed and Epileptic, Queen Square, and recounted by him in a recent lecture at the Polyclinic, represent troublesome pitfalls for the general practitioner, for even now students are not sufficiently catered for in regard to the diagnosis and treatment of nervous disorders, and comparatively few of those who embark on general practice attend the post-graduate courses of the special nerve hospitals. Work of this kind is likely to make a hospital as useful to the rank and file of the medical profession as the more scientific researches of its laboratories; and investigations that help to make clear to general practitioners the difficulties of diagnosis from the point of view of everyday routine are to be encouraged most warmly.

Professor Augustus D. Waller, M.D., F.R.S., whose name has been prominently mentioned in connection with the new Heart Hospital which was opened last Monday, January 12, in Westmoreland Street, is, of course, the distinguished director of the London University Physiological Laboratories. Dr. Waller holds the post of consulting physician to the hospital, to which he was elected many years ago, his pioneer investigations on electrocardiology being the link between his work and that of this special charity. Professor Waller has for the most part approached heart problems from the purely physiological point of view, leaving the clinical aspect of the subject to others. As lecturer at St. Mary's in the 'nineties the eminent physiologist was immensely popular with the large following of medical men and students who were attracted by his brilliant electro-physiological demonstrations.

Dr. Henry James Alford, M.D. (Lond.), who has just resigned the post of medical officer of health for Taunton rural district after forty years' service, is an old University College Hospital man, and took his doctor's degree in 1872.

